# Can Bioactive Compounds in Beetroot/Carrot Juice Have a Neuroprotective Effect? Morphological Studies of Neurons Immunoreactive to Calretinin of the Rat Hippocampus after Exposure to Cadmium

**DOI:** 10.3390/foods11182794

**Published:** 2022-09-10

**Authors:** Małgorzata Matysek, Edyta Kowalczuk-Vasilev, Radosław Szalak, Ewa Baranowska-Wójcik, Marcin B. Arciszewski, Dominik Szwajgier

**Affiliations:** 1 Department of Animal Anatomy and Histology, Faculty of Veterinary Medicine, University of Life Sciences, 12 Akademicka St., 20-950 Lublin, Poland; 2 Institute of Animal Nutrition and Bromatology, Faculty of Animal Science and Bioeconomy, University of Life Sciences, 13 Akademicka St., 20-950 Lublin, Poland; 3 Department of Biotechnology, Microbiology and Human Nutrition, Faculty of Food Science and Biotechnology, University of Life Sciences, 8 Skromna St., 20-704 Lublin, Poland

**Keywords:** cadmium, beetroot/carrot juice, calcium binding protein, calretinin, central nervous system, dementia

## Abstract

Cadmium ions (Cd^2+^) penetrate the blood–brain barrier and can, among other effects, influence intracellular calcium metabolism, leading to neurodegeneration. In the presented work, we estimated the effect of Cd^2+^ on the expression of calretinin in the neurons of the rat hippocampus and analyzed the reverse effect of freshly pressed beetroot/carrot juice in this context. In the 12-week lasting experiment, 32 8-week-old male Wistar rats were divided into four experimental groups (n = 8): the control group (C) received pure tap water; the Cd group (Cd)—received Cd^2+^ dissolved in tap water (5 mg Cd^2+^/kg b.w.); and two groups received beetroot/carrot juice: the BCJ group was administered only juice, and the Cd + BCJ group received juice with the addition of Cd^2+^ (5 mg Cd^2+^/kg b.w.). The exposition to low doses of Cd^2+^ caused a significant decrease in calretinin-immunoreactive (Cr-IR) neurons compared to the non-exposed groups. Moreover, the addition of Cd^2+^ to tap water reduced the numbers and length of Cr-IR nerve fibers. The negative effect of Cd^2+^ was significantly attenuated by the simultaneous supplementation of beetroot/carrot juice (Cd + BCJ). The study showed that the bioactive compounds in the beetroot/carrot juice can modulate Ca^2+^ levels in neurons, and thus, potentially act as a neuroprotective factor against neuronal damage.

## 1. Introduction

Cadmium (Cd^2+^) is a highly toxic heavy metal that is abundant in the environment. It enters the body through the respiratory or digestive tract, leading to excessive accumulation in internal organs, such as the kidneys, liver, or bones, increasing the risk of organ damage, osteoporosis, hypertension, or diabetes [1,2]. Cd^2+^ is also a known cause of neuroinflammation and dementia (including Alzheimer’s disease), as essentially pointed out in several excellent reviews (e.g., by Huat et al. [3]; Zhang et al. [4]). It is estimated that a significant part of Cd^2+^ present in the bloodstream crosses the blood–brain barrier and accumulates in various structures of the central nervous system (CNS) [5]. Within the CNS, the hippocampus is the target structure for Cd^2+^ toxicity. The hippocampus proper is a part of the limbic system. It plays an important role in the consolidation of information from short-term memory to long-term memory and in spatial navigation. On the one hand, it is a specific center of the brain’s memory and reasoning, and on the other, it is the area of the brain most affected by neurodegenerative diseases, of which dementia is the main symptom [6]. Cd^2+^ can also influence intracellular calcium (Ca^2+^) metabolism. Excessive accumulation of Ca^2+^ in nerve cells is responsible for the activation of processes that lead to neurodegenerative diseases, and ultimately to cell death [7]. Ca^2+^-binding proteins (CaBPs) provide an important first line of defense due to their ability to buffer incoming calcium, allowing the neurons to rapidly attain homeostasis. Moreover, it is known that Cd^2+^ is a strong calcium channel blocker—it inhibits Ca^2+^ uptake by cells, which disrupts the transmission of neuronal signals [8]. Calretinin (Cr) is one of the three main types of CaBPs present in inhibitory GABAergic neurons. The important function of Cr is the regulation of Ca^2+^ flow; it participates in synaptic plasticity and also influences the excitability of interneurons in the hippocampus by regulating other GABAergic neurons [9,10]. Studies on another heavy metal, Pb^2+^, have shown some promising effects in terms of the ability of beetroot juice to alleviate some of its toxic impact on the organism, particularly with regard to antioxidant and neurological functions [11,12,13]. 

The beet (*Beta vulgaris* L.) is a valuable source of unique natural compounds exerting antioxidant properties that may be beneficial to human health, as comprehensively presented in selected review papers, e.g., by [14] or [15]. It was confirmed that the consumption of beetroot may improve cerebral blood flow, and consequently, the cognitive function [16]. Beetroot extract showed anti-anxiety and antidepressant properties [17]. The carrot (*Daucus carota* L., *Apiaceae* family) is another highly appreciated, edible fleshy root, widely discussed in some excellent reviews, e.g., by Ahmad et al. [18] or Aćimović [19]. It is a source of α- and β-carotene, anthocyanins, lycopene, and phenolic acids [20], compounds known to have antioxidant properties (e.g., Mazewski et al. [21]). In our previous paper, we showed that beetroot/carrot juice was a rich source of bioactive compounds (mainly polyphenols, nitric pigments, and saponins). The juice revealed strong antioxidant activity, as confirmed using three experimental in vitro methods [22]. It has been reported before (e.g., in [3]), that Cd^2+^ can contribute to the development of Alzheimer’s disease. Although the presented paper focused in the first place on the ability of the juice to reduce the general toxicity of Cd^2+^ in the brain, a possible relationship between Cd^2+^ toxicity and acetylcholine-related enzymes was assessed as markers of cognitive function.

Based on the above, the present study aimed to evaluate the potential neuroprotective properties of bioactive compounds (present in beetroot/carrot juice), as a protective factor against neurodegenerative diseases, and also determine the effect of Cd^2+^ on neurons in the CNS, sensitive to Cd^2+^-induced memory disorders, with a particular focus on Cr immunoreactivity in rat hippocampal neurons. 

## 2. Materials and Methods

### 2.1. Preparation of Beetroot/Carrot Juice (BCJ)

The beetroot/carrot juice (BCJ) was prepared essentially as described in our previous work [22]. In short, freshly pressed juices were obtained from thoroughly washed vegetables (Opolski beetroot and Nantejska carrot; in tap water), using an Angel 750 low-pressure screw press (Angel Co. Ltd., Naarden, The Netherlands). A single raw juice blend was prepared by mixing the beetroot and carrot juices at a ratio of 4:1 *v*/*v* (with pH corrected to 4.0 by the addition of 0.25 g of citric acid per 100 mL of juice). The final juice was freshly prepared and administered to rats within 2 days. 

### 2.2. Qualitative Characteristics of Juice 

The composition of the juice, as well as its total polyphenolic content and antioxidant activity were given in our previous work [22]. The anticholinesterase activity of BCJ (against acetyl- and butyrylcholinesterase) was studied as described by Studzińska-Sroka et al. [23] using juice prepared as described above and not otherwise processed in any way prior to analysis. To check the pro-cognitive efficiency of the juice, the ability to inhibit acetyl- and butyrylcholinesterase activity was tested as described earlier [23], expressed as the equivalent concentration of donepezil, a well-known cholinesterase inhibitor. 

### 2.3. Animals

This experiment was approved by the 2nd Local Ethical Committee at the University of Life Sciences in Lublin, Poland (ref. no. 105/2015). Thirty-two (n = 32) male, 8-week-old Wistar rats were kept in single cages under a 12 h light–dark cycle with the light on at 06:00 a.m., at a room temperature of 21 ± 1 °C and a relative humidity of 55 ± 10%, with the cages ventilated every 4 min. The rats received the standard commercial rodent food (LSM; Agropol, Motycz, Poland; containing 12.06 MJ/kg ME (metabolic energy); crude protein: min. 16.00%; crude fat: min. 2.8%; crude ash: max. 7.00%; crude fiber: max. 5.00%; calcium min. 1.10%; phosphorus min. 0.70%; sodium max. 0.22 %; and vit. A: 8000 IU/kg; Vit. D3: 1000 IU/kg; Vit. E: 50 mg/kg) in the amount of 210 g per rat per week. Cadmium chloride of very high purity (99.999%) was used (Sigma-Aldrich, Poznań, Poland, cat. no 439800). The animals were acclimatized to the laboratory for at least one week before being used in the experiment. The rats were randomly divided into four experimental groups (n = 8): the control group (C) received 100 mL of tap water; the Cd group (Cd) received Cd^2+^ dissolved in 100 mL tap water/day (equivalent to 5 mg Cd^2+^/kg b.w.); and two groups received beetroot/carrot juice: the BCJ group was administered only 100 mL of juice per day, and the Cd + BCJ group received juice with the addition of Cd^2+^ ions (Cd^2+^ dissolved in 100 mL juice/day (equivalent to 5 mg Cd^2+^/kg b.w.). The experimental dose of Cd^2+^ was based on literature data [24,25,26]. The feed and fluid intake was controlled daily. The experiment lasted for 12 weeks.

### 2.4. Experimental Procedures and Analyses 

After euthanasia (decapitation), the rats’ brains were stored in 10% buffered formalin (pH = 7) for 12 h at 4 °C, hydrated in decreasing concentrations of ethyl alcohol, and embedded in paraffin blocks in accordance with the previously described method [27]. Briefly, the paraffin blocks were cut into 5 μm-thick sections which were placed on silanized glass-slides (SuperFrostPlus, Thermo Fisher Scientific, Braunschweig, Germany). To block the endogenous peroxidase activity, the sections were chilled and washed in 3% hydrogen peroxidase (20 min). The slides were then flushed twice with PBS (pH 7.4) (15 min each time) and incubated in 2.5% normal goat serum (ImPRESS^TM^; MP-7451, Vector Labs, Burlingame, CA, USA) at room temperature (RT) for 20 min. The sections were incubated for 24 h at 4 °C with primary monoclonal mouse antibodies raised against Cr (1:2000; C7479, Sigma, Taufkirchen, Germany). The next day, the slides were washed in a washing buffer (2 × 15 min) and covered with anti-mouse/rabbit Ig (ImPRESS^TM^; MP-7500 Vector Labs, Newark, CA, USA) for 1 h. The specificity of the antibodies used was verified using a negative control in which primary antibodies were replaced with the same concentrations of appropriate non-immune IgG. 3,3′-diaminobenzidine chromogen (ImmPACT^TM^DAB, SK-4105, Vector Labs, Newark, CA, USA) was used to visualize the primary antisera. A working solution of DAB was applied onto the sections, and the process was monitored under a light microscope. Finally, the slides were rinsed with distilled water. Moreover, counterstaining (for 20 min) with Mayer’s hematoxylin was performed. After washing in distilled water, the sections were dehydrated in increasing concentrations of ethyl alcohol, cleared in xylene, mounted in DPX (Sigma-Aldrich, St. Louis, MO, USA), and cover slipped. The slides were viewed under a light microscope (Axiolab, Zeiss, Jena, Germany) connected to a digital camera (Olympus Color View III, Tokio, Japan). From each animal, approx. 25–30 sections immunostained for Cr were examined. The Cr-IR neurons were assessed by analyzing and counting no less than one hundred neurons immunoreactive (IR) to Cr in the CA1 field of the hippocampus of each group (the control and experimental) using Cell D software (Olympus, Tokio, Japan). Image J software (ImageJ 1.53 k; National Institute of Health, Bethesda, MD, USA) was used to quantify and statistically compare the length of Cr-IR nerve fibers. At least two independent observers were involved in quantification analyses, and the results obtained by them were averaged. 

### 2.5. Statistical Analysis

The collected data were analyzed with Statistica software ver. 13.1 ((StatSoft, Kraków, Poland). Normality was assessed using the Kolmogorov–Smirnov test, and Levene’s homogeneity of variance test was applied to examine the equality of variances. One-way ANOVA and Tukey’s post-hoc tests were performed to compare all the experimental groups individually, whereas the two-way ANOVA was used to determine the impact of experimental factors: Cd^2+^ exposure and beetroot/carrot juice treatment and their interaction. Significant differences between the groups were identified at *p* ≤ 0.05 and *p* ≤  0.01.

## 3. Results

### 3.1. Anticholinesterase Activity of the Studied Juice 

In our previous paper [22], we presented the composition of BCJ (using high-resolution, qualitative, and quantitative HPLC-ESIQTOF- MS) and identified the main bioactive components present (betanin, isobetanin, vulgaxanthin I and II, indicaxanthin, neobetanin, dexarboxyneobetanin, and decarboxybetanin). The juice showed significant total polyphenolic content, high antioxidant activity (tested using three experimental models), and positive effects towards human colon epithelial normal and cancer cells [22], so it was a promising research material in the context of the presented work. In the present work, we tested the ability of the juice to decrease the activity of acetyl- and butyrylcholinesterase and we report that the activity towards both enzymes was high (equal to donepezil applied at the concentration of 0.05 ± 0.00 µg/mL and 0.09 ± 0.01 µg/mL, respectively). 

### 3.2. Cr Expression in the CA1 Field of the Hippocampus

**Figure 1:**
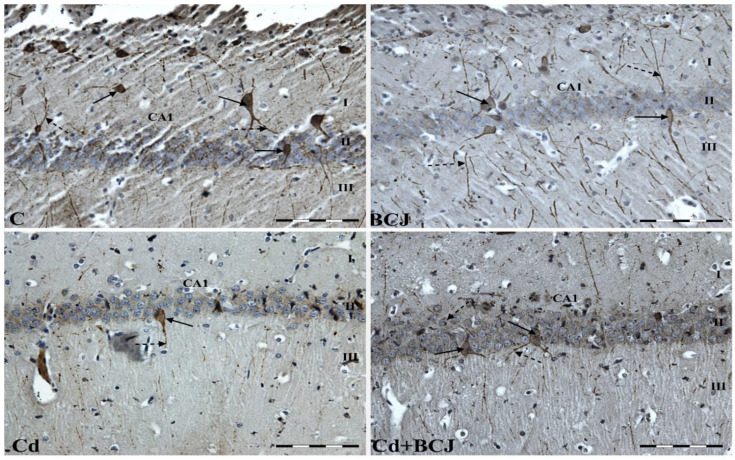
Cr-IR neurons and Cr-IR fibers in the hippocampal CA1 fields of experimental rats’ brains in the respective groups: C—the control group received 100 mL of tap water; Cd group—received Cd^2+^ dissolved in 100 mL tap water/day (equivalent to 5 mg Cd^2+^/kg b.w.); BCJ group—received 100 mL of juice per day; Cd+BCJ group—received juice with the addition of Cd^2+^ ions (Cd^2+^ dissolved in 100 mL juice/day (equivalent to 5 mg Cd^2+^/kg b.w.); I: the marginal layer, II: the pyramidal layer, III: the multiform layer; the arrows indicate Cr-IR neurons (solid one) and Cr-IR fibers (dotted one) of the hippocampus. Scale bars = 20 μm.

**Figure 2:**
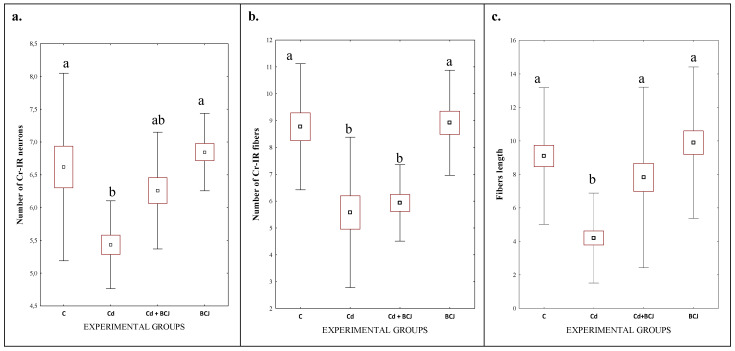
The figures present: the average numbers of Cr-IR neurons (**a**) and the average number of Cr-IR fibers (**b**) in the CA1 fields of rat hippocampus and the average length of nerve fibers (**c**). The data are expressed as means ± SEM (standard error of the mean; box) and standard deviation (whiskers); ^a,b^—different letters indicate significant differences between the experimental groups at *p* ≤ 0.01.

The hippocampus consists of the Ammon’s horn (CA1–CA3) and the dentate gyrus (DG), but in the present study, we focused on the CA1 field of the rat hippocampus due to the high sensitivity of the nerve cells in this area to damage, as compared to CA2 and CA3 fields of the hippocampus [28]. The dorsal portion of the hippocampus was examined. In all the studied groups, multiform (oval, round, triangular, and fusiform) Cr-IR neurons were observed, unevenly distributed in all layers (the marginal, pyramidal, and multiform) of the CA1 field of the rat hippocampus. The neurons were characterized by the presence of cytoplasmic and nuclear reactions (Figure 1). The average numbers of Cr-IR neurons in the CA1 field of the rat hippocampus were estimated at 6.62 ± 0.72 in the control group, while the analogous neuronal populations in the BCJ group were calculated at 6.85 ± 0.30 (Figure 2a). There were no statistically significant differences in the mean numbers of Cr-IR neurons between the control group receiving tap water and the BCJ group (Figure 2a). In both cases, the neurons showed intense (+++) or moderate (++) nuclear/cytoplasmic reactions to Cr (Figure 1 C and BCJ). 

Although collectively in both groups exposed to Cd^2+^, a decrease in the average number of Cr-IR neurons was stated in comparison to non-exposed groups (*p* < 0.001; Figure 2a; Table 1), and a statistically significant decrease (*p* ≤ 0.01) in the average number of Cr-IR neurons was only observed in the group receiving Cd^2+^ with tap water (Cd) as compared to control group (6.62 ± 0.72 vs. 5.43 ± 0.33, respectively) (Figure 2a). On the other hand, a positive trend was stated in case of the BCJ treated group. The mean number of Cr-IR neurons was higher in the brains of rats receiving Cd^2+^ dissolved in beetroot/carrot juice (Cd + BCJ group) as compared to the Cd group, although the differences were not confirmed statistically (Figure 2a). BCJ treatment significantly affected the numbers of Cr-IR neurons as compared to non-treated groups (6.55 ± 0.47 vs. 6.03 ± 0.82; *p* = 0.025; Table 1). In the Cd^2+^-treated group (Cd), the nuclear/cytoplasmic reaction was weak (+) and individual Cr-IR neurons were located only in the layer of pyramidal cells of the CA1 fields (Figure 1 Cd). In contrast, in the Cd^2+^ + BCJ group, weak (+) to moderate (++) nuclear/cytoplasmic reactions in Cr-IR neurons were recorded, and neurons IR to Cr were present in all layers of the hippocampal CA1 fields (Figure 1 Cd + BCJ). 

The immunoreactivity to Cr was also observed in the nerve fibers of the hippocampal CA1 field. In the control and BCJ groups, the reaction to Cr in the nerve fibers was intense (+++) or moderate (++) (Figure 1 C and BCJ). The Cr-IR nerve fibers were numerous (the average numbers of Cr-IR nerve fibers were 8.77 ± 1.17 in the control group and 8.92 ± 0.98 in the BCJ group; Figure 2b) and long (9.10 µm and 9.89 µm, respectively; Figure 2b). There were no statistically significant differences in terms of the mean number of nerve fibers between those groups (Figure 2b). This is in contrast to the Cd^2+^-treated groups (Table 1). In the Cd^2+^-treated group (Cd), receiving Cd^2+^ dissolved in tap water, Cr-IR nerve fibers were less numerous (5.58 ± 1.40; Figure 2b), singular, with a weak (+) Cr-IR reaction (Figure 1). Moreover, in many areas of the CA1 field, no Cr-IR nerve fibers were observed (Figure 1 Cd). Exposition to Cd^2+^ had a significant impact on both the number, as well on the length, of the nerve fibers (*p* < 0.0001; Table 1). The length of the nerve fibers in the Cd^2+^ group was the shortest among all of the experimental groups (Figure 2c; Table 1). The supplementation of the BCJ juice attenuated the negative impact of cadmium ions on the length of the nerve fibers (4.20 µm in Cd groups vs. 7.82 µm in Cd+BCJ group; Figure 2c; *p* = 0.002; Table 1). The statistical analysis confirmed a significant interaction between both experimental factors (Cd and BCJ addition) on the length of the nerve fibers (*p* = 0.044; Table 1).

## 4. Discussion

The presented results show that chronic exposure to even low doses of Cd^2+^ can be associated with neurodegenerative disorders characterized by memory impairment or dementia. As it reaches the brain, Cd^2+^ inhibits neurogenesis, including in the hippocampus [29], while also producing free radicals that can damage neurons [30]. Based on the findings reported by Lopez et al. [31], it can be argued that Cd^2+^ affects the morphology of nerve cells—the changes mainly concern nerve projections (axons and dendrites), which completely disappear after prolonged exposure to Cd^2+^. Likewise, the results of the present study show that there is a relationship between the consumption of Cd^2+^ in tap water and a weak Cr immunoreactivity observed in the nerve fibers of the hippocampal CA1 field. As compared to the control group, the groups of rats receiving Cd^2+^ (Cd and Cd + BCJ) had significantly fewer nerve fibers. Not only was the number of nerve fibers lower, the analysis of images confirmed that the nerve fibers were also significantly shorter. There were no statistically significant differences in the average number of nerve fibers between the groups receiving Cd^2+^, irrespective of the solvent used (Figure 2c; Table 1).

In the present study, Cr immunoreactivity in the CA1 field of the rat hippocampus was assessed to investigate the effect of beetroot/carrot juice on neurons present in the CNS structure sensitive to memory disorders due to administration of Cd^2+^. Previously, it was shown that beetroot consumption may improve the cognitive functions of the brain by facilitating better cerebral blood flow [32]. Olasehinde et al. [33] observed an improvement of cognitive functions (decreased by scopolamine) in rats receiving beetroot powder (2 and 4% in feed). In another study, beetroot extract (100 μg d.m. mL^−1^) lowered the activity of acetylcholinesterase (by 93.3% as compared to 94.2% observed for the standard enzyme inhibitor donepezil at the same concentration) [34]. The consumption of raw beet (100 g a day for 8 weeks) significantly improved antioxidant activity as well as cognitive functions in a group of patients suffering from type II diabetes [35]. It was previously shown that beetroot can exert a positive action against toxic heavy metal ions in the context of neurodegeneration and cognitive deficits. The administration of lead (Pb acetate, 40 mg/kg b.w.) intensified lipid oxidation and reduced glutathione levels and antioxidant capacity in cerebral tissues of rats. However, the administration of beet juice (8 mL kg^−1^ b.w.) increased the level of glutathione levels (15 vs. 25 mg GSH/g cerebral tissue). Moreover, acetylcholinesterase activity in the cerebral tissue was elevated by approximately 15% as compared to the group receiving Pb only [12]. Similarly, acetylcholinesterase activity levels were reduced in the blood of farmers who consumed a beet-based beverage for two months (2 months, 2 × 500 mL daily) [13].

Calcium is an omnipresent intracellular ion that acts as a signaling mediator in many cellular processes, including proliferation, differentiation, and cell survival/death. It is also involved in long-term processes such as memory acquisition, which are mediated by the interaction of Ca^2+^ with intracellular CaBPs [36]. Cr is a good neuronal marker due to its intracellular Ca^2+^ buffering properties. It is probable that a change in the level of CaBPs or their modification may lead to an impairment of neuronal calcium homeostasis and, consequently, cause pathological reactions and even cell death. Such changes may result from diabetes mellitus, neurodegenerative diseases, but also from the toxic effects of heavy metals. According to Xu et al. [37] and Yuan et al. [38], exposure to cadmium disrupts intracellular Ca^2+^ homeostasis, thereby inducing apoptotic morphological changes in neurons. In the present study, Cr-IR neurons were observed in all layers of the CA1 field of the rat hippocampus. The mean number of Cr-IR neurons in the control group and the group receiving beetroot/carrot juice was similar; no statistically significant differences were found. In both groups receiving Cd^2+^, a decrease in the average number of Cr-IR neurons was shown as compared to the control group; however, in the group receiving Cd^2+^ with tap water the number of Cr-IR neurons was lower than in the group receiving Cd^2+^ dissolved in beetroot/carrot juice, which confirms the protective capacity of bioactive components present in the beetroot. The study also confirmed that Cd^2+^ intake is associated with the weak response of Cr-expressing hippocampal neurons. Accordingly, exposure to Cd^2+^ reduces the number of Cr-expressing nerve cells in the hippocampus.

## 5. Conclusions

The obtained results may suggest that chronic exposure to even low doses of Cd^2+^ can be associated with neurodegenerative disorders characterized by memory impairment or dementia. Cd^2+^ had a significant impact on the number of neurons, and also on the morphology of nerve fibers, as well as the immunoreactivity to Cr in rat hippocampal neurons, which impaired intracellular Ca^2+^ homeostasis.

However, the negative impact of Cd^2+^ was probably reduced with the inclusion of beetroot/carrot juice. The beneficial effects of beetroot/carrot juice may be related to the action of bioactive compounds that induce an increase of intracellular Ca^2+^ in the hippocampal neurons, thus preventing the toxic influence of heavy metals on the CNS structures and a protective factor against neurodegenerative diseases. The above data may suggest that the regulation of Cd-induced Ca^2+^ homeostasis may be a good strategy in the prevention of diseases affecting the CNS structures and provide the basis for further research into the possible neuroprotective role of bioactive compounds.

## Figures and Tables

**Table 1:** The impact of experimental factors: Cd exposition and BCJ treatment on average numbers of Cr-IR neurons and Cr-IR nerve fibers and length of nerve fibers (µm) in the CA1 field of rat hippocampus in the experimental groups.

Parameter	Cd Exposition		BCJ Supplementation		Two-Way ANOVA		
					Impact of		
	−	+	−	+	Cd Exposition	BCJ Treatment	Cd × BCJ
No of Cr-IR neurons	6.73 ^a^ ± 0.53	5.85 ^b^ ± 0.57	6.03 ^B^ ± 0.82	6.55 ^A^ ± 0.47	<0.001	0.025	0.178
No of Cr-IR fibers	8.85 ^a^ ± 1.02	5.76 ^b^ ± 1.06	7.18 ± 2.08	7.43 ± 1.77	<0.00001	0.620	0.835
Length of nerve fibers	9.50 ^a^ ± 2.13	6.01 ^b^ ± 2.78	6.65 ^b^ ± 3.02	8.86 ^a^ ± 2.64	<0.0001	0.002	0.044

Explanations: The data are expressed as means ± SD (standard deviation); ^a,b^—values in the rows with different letters differ significantly at *p* ≤ 0.01; ^A,B^—values in the rows with different letters differ significantly at *p* ≤ 0.05.

## Data Availability

Data is contained within the article.
